# Gastric bronchogenic cyst presenting as a submucosal mass: a case report

**DOI:** 10.1186/1752-1947-6-262

**Published:** 2012-08-31

**Authors:** Hassan Seddik, Tarik Adioui, Fadoua Rouibaa, Fatima Zohra El Hamdi, Aziz Aourarh, Mohammed Mahi, Ahmed Benkirane, Aziz Zentar

**Affiliations:** 1 Department of Gastroenterology II, Mohamed V Teaching Military Hospital, Rabat 10100, Morocco; 2 Department of Gastroenterology I, Mohamed V Teaching Military Hospital, Rabat 10100, Morocco; 3 Department of Radiology, Mohamed V Teaching Military Hospital, Rabat 10100, Morocco; 4 Department of General Surgery I, Mohamed V Teaching Military Hospital, Rabat 10100, Morocco

**Keywords:** Bronchogenic cyst, Stomach

## Abstract

**Introduction:**

Bronchogenic cysts are developmental anomalies of the primitive foregut which mostly occur in the lung. Gastric bronchogenic cysts are extremely rare; few cases have been reported in the literature and the diagnosis was often made following surgical resection.

**Case presentation:**

A 40-year-old North African man was admitted to our hospital with a gastric submucosal mass. An endoscopic ultrasound revealed a unilocular cystic mass located in the muscular layer. Its content was echogenic suggestive of mucus. Magnetic resonance imaging confirmed the liquid nature of the cyst and showed a high ratio of proteins. Based on these observations, the diagnosis of bronchogenic cyst was confirmed. An endoscopic monitoring was decided rather than surgery because of the small size of the cyst and the absence of symptoms.

**Conclusion:**

Although gastric bronchogenic cysts are rare, they should be well known and considered in all differential diagnoses of gastric tumors. We report a new case of gastric bronchogenic cyst and highlight the contribution of morphological tests that currently allow a non-invasive diagnosis.

## Introduction

Bronchogenic cysts are congenital anomalies evolving through the ventral foregut. They are the result of an autonomous and delayed development of a cell bud from the tracheobronchic tree. Bronchogenic cysts are divided into thoracic and abdominal cysts [1,2]. Abdominal bronchogenic cysts are rare, especially those located exclusively within the confines of the gastric wall. Few cases have been reported in the literature and the diagnosis was often made upon surgical resection [3]. We report a new case of gastric bronchogenic cyst and highlight the contribution of morphological tests that currently allow a non-invasive diagnosis.

## Case presentation

**Figure 1:**
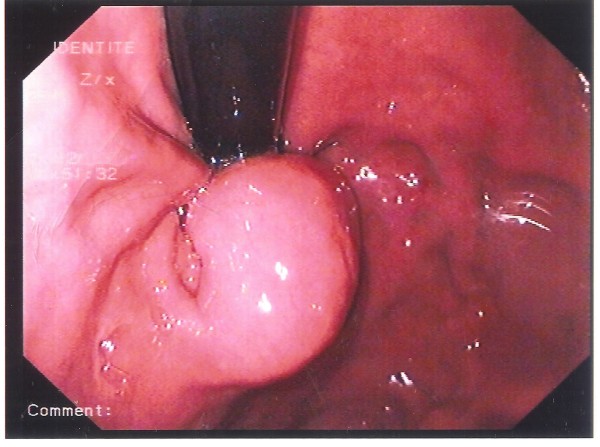
An esophagogastroduodenoscopy showed a submucosal mass in the patient’s juxtacardial stomach.

**Figure 2:**
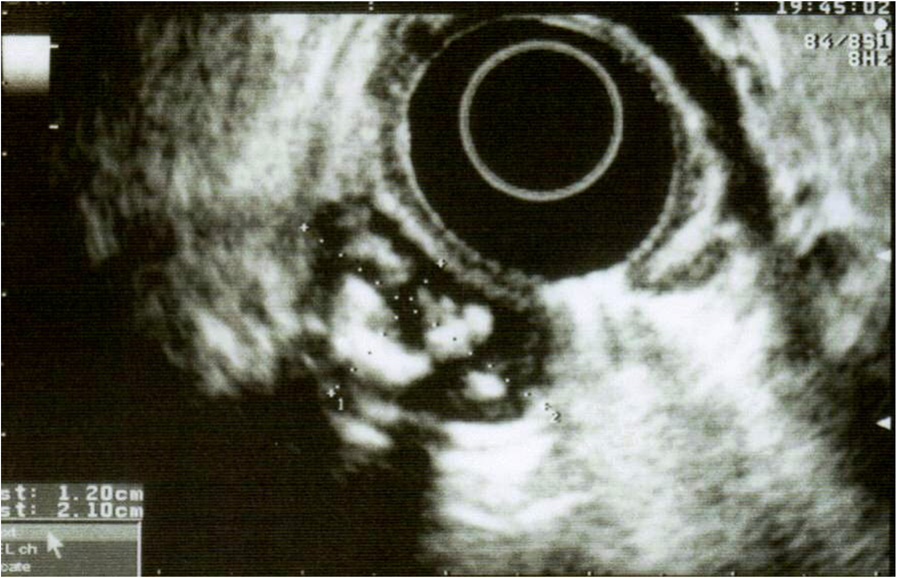
An endoscopic ultrasound showed a unilocular cystic lesion, developed in the muscularis, containing echogenic images.

A 40-year-old North African man was admitted to our hospital with an epigastric pain that had lasted for 6 months without dysphagia or hematemesis. His overall health condition was good and a clinical examination did not reveal anything notable except that the patient was slightly overweight. A gastroscopy revealed a submucosal mass, which was 2cm in diameter, in the juxtacardial stomach (Figure 1). A histological examination did not help the diagnosis and revealed only *Helicobacter pylori* gastritis. An endoscopic ultrasound (EUS) confirmed the submucosal location of the lesion. It showed an image of a unilocular cyst containing echoic spots suggestive of mucus. This lesion was located in the muscular layer 45cm from the dental arches and it measured 15×19mm (Figure 2). Magnetic resonance imaging (MRI) denoted a homogeneous hypersignal in sequences T1 and T2, with no enhancement after injecting the contrast agent, confirming the liquid nature of the cyst. It also showed a hypersignal T1 indicating a high ratio of proteins in the cyst content. Hydatic serology was negative. Based on the results of the morphological examinations, especially the MRI and EUS, the diagnosis of bronchogenic cyst was retained. We submitted the patient to *H. pylori* eradication therapy which stopped his pain. We decided then to merely monitor the cyst due to its small size and the absence of symptoms.

Endoscopic controls realized during the following 3 years did not show any changes in the lesion aspect.

## Discussion

Bronchogenic cysts located in the gastric wall are most often asymptomatic and discovered incidentally during a radiological or endoscopic test. The risk of further complication (intracystic bleeding, superinfection, compression) is not known in this location [3] whereas there is a high risk of further complications with pulmonary bronchogenic cysts. The morphology of gastric bronchogenic cysts may be misleading. In endoscopy they look like external compressions or submucosal masses suggestive of stromal tumor especially if located in the fundus. Usually, a histological examination also does not help the diagnosis. A computed tomography scan shows a round or oval-shaped mass, well-circumscribed and thin-walled, with fluid density and no enhancement after injecting the contrast agent. However, the lesion can be misdiagnosed as a solid mass in cases in which it contains thick mucinous and proteinaceous secretions [4]. An EUS test shows a hypoechoic or anechoic, well-circumscribed, round or oval lesion which is located in the fourth hypoechoic layer of the gastric wall. Despite a good performance in terms of diagnosing gastric bronchogenic cysts, EUSs can sometimes be limited by the variability of the components of the cyst content. In the presence of thick fluid content, an EUS cannot exclude an encysted stromal tumor, which can also be located in the fourth hypoechoic layer and may contain intratumoral cysts. Some authors [5,6] suggest searching ciliated cells via a cytological study performed by an endosonographically-guided fine-needle aspiration. However, this aspiration can be challenging due to the thick content of the cyst and it may expose to superinfection of the cyst content [3,7]. The MRI was useful in the diagnosis [4] by showing homogeneous hypersignal in sequences T1 and T2, with no enhancement after injecting the contrast agent, confirming the liquid nature of the cyst. In our patient’s case, confronting the results of the different morphological examinations allowed us to retain our gastric bronchogenic cyst diagnosis and we decided a mere endoscopic monitoring was sufficient treatment.

## Conclusion

Although gastric bronchogenic cysts are rare, they should be well known and considered in all differential diagnoses of gastric tumors. We report a new case of gastric bronchogenic cyst and highlight the contribution of morphological tests that currently allow a non-invasive diagnosis.

## Consent

Written informed consent was obtained from the patient for publication of this manuscript and accompanying images. A copy of the written consent is available for review by the Editor-in-Chief of this journal.

## Competing interests

The authors declare that they have no competing interests.

## Authors’ contributions

HS and TA evaluated the patient and were major contributors to the writing of the manuscript. AA and FE performed the gastroscopy. FR performed the EUS. MM analyzed and interpreted the data from the MRI. AB and AZ reviewed the manuscript. All authors read and approved the final manuscript.
